# Supplementary material for: Complete Chloroplast Genome and Phylogenomic Analysis of Davallia trichomanoides (Polypodiaceae)
Source: Genes (Basel). 2025 Nov 1;16(11):1310. doi: 10.3390/genes16111310 (PMC12652714; doi:10.3390/genes16111310)
Supplement: Supplementary file 1 [file genes-16-01310-s001.zip › Figure S1.pdf]

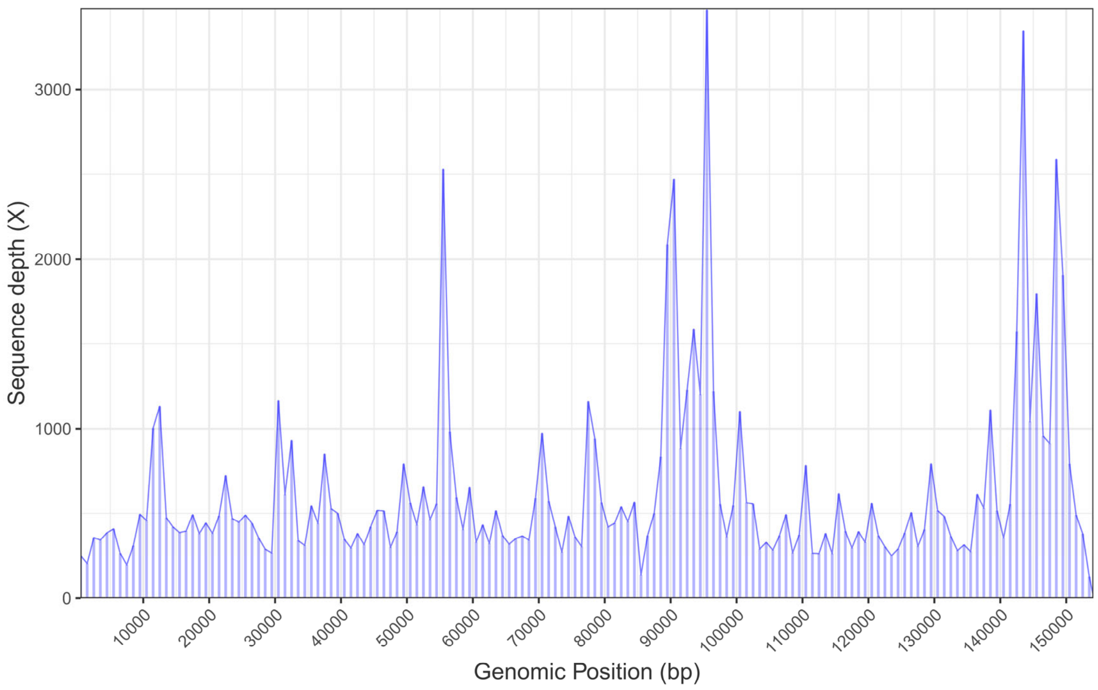

Figure S1. The distribution of chloroplast genome sequencing depth for *Davallia trichomanoides* is depicted in the graph, with the horizontal axis representing genomic position and the vertical axis indicating sequencing depth.
