# Supplementary material for: Complete Chloroplast Genome and Phylogenomic Analysis of Davallia trichomanoides (Polypodiaceae)
Source: Genes (Basel). 2025 Nov 1;16(11):1310. doi: 10.3390/genes16111310 (PMC12652714; doi:10.3390/genes16111310)
Supplement: Supplementary file 1 [file genes-16-01310-s001.zip › Table S1,2.pdf]

Table S1. All clean data information

	Davallia trichomanoides
Total Reads Count (#)	93481172
Total Bases Count (bp)	13927284473
Average Read Length (bp)	149
Q20 Bases Count (bp)	13765366554
Q20 Bases Ratio (%)	98.84%
Q30 Bases Count (bp)	13362182445
Q30 Bases Ratio (%)	95.94%
GC content (%)	56.99%

Table S2. Coverage summary

	Value	Percent of genome (%)
Genome length (bp)	154217	100.00
Mapped reads (count)	647533	
Mapped bases (bp)	82606670	
Mean depth (×)	535.652	
Covered ≥ 1× (bp)	154217	100.00
Covered ≥ 10× (bp)	154202	99.99
Covered ≥ 50× (bp)	154106	99.93
Covered ≥ 100× (bp)	152802	99.08
Covered ≥ 200× (bp)	142508	92.41
Overall covered (≥1×)		100.00
